# The neurogenic elbow disharmony loop: a pathophysiologic model for chronic elbow pain supported by one hundred and fifty seven radial tunnel decompressions

**DOI:** 10.1007/s00264-026-06929-6

**Published:** 2026-06-29

**Authors:** Elisabet Hagert, Ulrika Jedeskog, Enejd Veizi

**Affiliations:** 1 Dept of Surgery, Qatar Orthopaedic and Sports Medicine Hospital, Doha, Qatar; 2 College of Medicine, QU Health, Qatar University, Doha, Qatar; 3 Idrottskliniken Rehab, Solna, Sweden; 4 Ankara Yildirim Beyazit University, Faculty of Medicine, Ankara, Turkey

**Keywords:** Radial tunnel syndrome, Lacertus syndrome, Tennis elbow, Lateral epicondylitis, Disharmony loop

## Abstract

**Introduction:**

Radial tunnel syndrome (RTS) remains an overlooked cause of chronic elbow pain. Clinical overlap with lateral epicondylitis, and the coexistence of other nerve entrapments contribute to diagnostic uncertainty. This study aimed to characterise the clinical presentation, and concomitant pain/nerve entrapment patterns following surgical decompression of posterior interosseous nerve (PIN) compression, and to explore the Neurogenic Elbow Disharmony Loop (NEDL) as a hypothesis-generating model for refractory elbow pain.

**Methods:**

A retrospective review of 157 operated arms in 143 patients who underwent PIN decompression between 2012–2021 was performed. RTS was diagnosed clinically using a triad consisting of extensor carpi ulnaris (ECU) weakness, a positive sensory collapse test, and focal tenderness at the arcade of Frohse. Demographics, pain distribution, concomitant procedures, Quick/Work-DASH scores, postoperative visual analogue scale (VAS) scores, and patient satisfaction were analysed.

**Results:**

The mean age was 45.9 years, and the majority (66.9%) had previously been diagnosed with lateral epicondylitis. Referred symptoms were common, involving the hand (45%), shoulder (38%), wrist (28%), and neck (14%). Concomitant lacertus syndrome requiring surgical release was identified in 72.6% of arms. Return of ECU power was confirmed in all operated arms intraoperatively or before discharge. Mean pre-/postoperative Quick-/Work-DASH scores improved from 43.4 to 11.0 (*p* < 0.001), and 56.2 to 11.2 (*p* < 0.001), respectively. Mean postoperative VAS scores were 1.8 (pain), 1.1 (numbness), and 8.5 (satisfaction), with 83% of patients reporting good/excellent outcomes.

**Conclusion:**

Surgery with PIN decompression resulted in significant functional improvement and high patient satisfaction. The high prevalence of concomitant lacertus syndrome and the bidirectional distribution of referred symptoms support a neurogenic–biomechanical interpretation of chronic elbow pain. The NEDL is proposed as a hypothesis-generating framework that may guide the evaluation and treatment of patients with refractory elbow pain.

## Introduction

Chronic lateral elbow and proximal forearm pain are both frequently attributed to tendinopathy, overuse, or referred cervical and shoulder pathology [1–3]. Peripheral nerve compression represents an important and often under-recognized contributor to nontraumatic upper-extremity pain [3, 4]. This is particularly relevant in high-use arms, where repetitive loading, sport- or work-specific biomechanics may predispose the median, radial, ulnar, and other upper-limb nerves to dynamic entrapment [5]. Radial tunnel syndrome (RTS), involving compression of the posterior interosseous nerve (PIN) within the radial tunnel, represents an important yet frequently overlooked cause of chronic lateral elbow and proximal forearm pain.. Symptoms often overlap substantially with lateral epicondylitis but also because physical examination, imaging, electromyography, and nerve conduction studies have limited ability to reliably localize compression or guide surgical decision-making [2]. Importantly, PIN compression at the arcade of Frohse commonly presents with exertional or nocturnal dorso-proximal forearm pain and is frequently misdiagnosed as a “tennis elbow”. Although traditionally considered a motor nerve entrapment, the PIN also carries sensory, proprioceptive, and nociceptive afference from the dorsal wrist, providing a neuroanatomical basis for symptoms that extend beyond isolated motor dysfunction and potentially explaining the broad symptom distribution frequently observed in these patients. [4].

The diagnostic challenge posed by RTS may be partly explained by the physiology of low-grade chronic nerve compression. Mild or dynamic compression may reduce endoneurial circulation and produce neural oedema, pain, and subtle weakness without axonal degeneration. The absence of muscle atrophy and consistent abnormalities on electrodiagnostic studies further complicates diagnosis [4]. Contemporary nerve-injury frameworks have expanded this concept through the description of ischaemic neurapraxia, or Sunderland 0 injury, in which electrodiagnostic studies may be normal because they measure axonal loss and demyelination, but not chronically ischaemic axons [6, 7]. In this setting, rapid restoration of muscle strength following decompression may reflect resolution of an ischaemic conduction block rather than axonal regeneration [7, 8]. The diagnosis of proximal upper-limb nerve entrapments has therefore increasingly relied on a structured clinical triad consisting of manual muscle testing to identify weakness in muscles distal to the suspected compression site, focal tenderness or Tinel’s sign at the level of entrapment, and sensory (scratch) collapse testing (SCT) [4, 9]. Although the SCT remains controversial, recent physiologic studies suggest that it may represent a protective reflex mediated through reticulospinal pathways activated by afferent input from an injured nerve [10, 11].

Taken together, these observations suggest that chronic lateral elbow pain should not be viewed solely through the lens of tendinopathy or isolated nerve compression, but rather as a potential manifestation of interacting neurogenic and biomechanical factors [4, 12]. Lacertus syndrome, a dynamic proximal median nerve entrapment at the level of the lacertus fibrosus, typically presents with loss of hand strength, loss of endurance, and forearm pain, while sensory symptoms may be delayed due to the fascicular arrangement of the median nerve at the elbow [9, 12]. These deficits may impair grip, pinch, flexor-pronator function, and proprioceptive control, potentially promoting compensatory wrist extension and increased lateral elbow loading. In parallel, RTS/PIN compression may amplify dorsal forearm pain, wrist and hand symptoms, and altered upper-limb proprioception; multi-site compression and muscle imbalance may further perpetuate symptoms along the kinetic chain [3]. “Disharmony loop” models like these have been proposed to explain the focal neurogenic dysfunction generating self-perpetuating pain, altered posture, and secondary neuropathic lesions across the upper limb [13, 14]. These observations raise the possibility that chronic lateral elbow pain may represent a disorder of interacting nerve compressions and compensatory biomechanical adaptations rather than a single isolated pathology.

Building on these concepts, this study proposes a Neurogenic Elbow Disharmony Loop in which repetitive stress, dynamic median nerve compression at the lacertus fibrosus, compensatory wrist extension, lateral elbow overload, and PIN compression interact to create a self-perpetuating neurogenic–biomechanical pain pattern. To date, the coexistence of PIN compression, lacertus syndrome, and widespread referred symptoms has received little attention in the literature. Although RTS and lacertus syndrome are increasingly recognized as distinct clinical entities, their potential interaction and contribution to chronic lateral elbow pain have not been systematically investigated. Therefore, the purpose of this study was to characterize the clinical presentation, referred pain distribution, concomitant nerve entrapment patterns, and patient-reported outcomes following surgical decompression of PIN compression at the Arcade of Frohse, and to explore the Neurogenic Elbow Disharmony Loop (NEDL) as a unifying, hypothesis-generating model for refractory lateral elbow and proximal forearm pain.

## Methods

This study involving human participants was in accordance with the ethical standards of the 1964 Helsinki Declaration and its later amendments. Ethics committee approval and due consent were also obtained. A retrospective study of prospectively collected data was conducted on the patient registry from June 2012 to June 2021. Patients were included for final evaluation if they had undergone surgical decompression of the posterior interosseous nerve at the level of the arcade of Frohse.

Medical charts were reviewed, and data on sex, age, occupation, hand dominance, prior diagnosis of “tennis elbow”, pain distribution, and surgical treatments were collected. Available pre-and postoperative quick-DASH (Disability of the Arm Shoulder Hand questionnaire) with work sub-scores and postoperative Visual Analogue Scale (VAS) of pain, numbness, and subjective satisfaction with the surgical outcome were analyzed.

### Radial tunnel syndrome diagnosis

The diagnosis of RTS was based on thorough patient history and clinical examination, including the previously described clinical triad of upper-extremity nerve compression: manual muscle testing revealing weakness in the extensor carpi ulnaris (ECU), a positive sensory (scratch) collapse test (SCT), and focal pain at the level of the arcade of Frohse in the proximal forearm [4]. Possible concomitant lacertus syndrome was diagnosed according to the established clinical triad for proximal median nerve entrapment at the elbow: weakness on manual muscle testing of the flexor carpi radialis (FCR), flexor pollicis longus (FPL), and flexor digitorum profundus to the index finger (FDP II) and a positive SCT over the median nerve at the level of the lacertus fibrosus with pain on compression of the median nerve at this level [12, 15]. Concomitant lateral epicondylopathy was defined by positive provocative manoeuvres and point-of-care ultrasound confirming tendinopathy of the common extensor tendon origin at the lateral epicondyle. Electromyographic studies were rarely implemented in the diagnostic workup and in cases where performed, were always negative/inconclusive.

### Surgical technique

The PIN was consistently released using a dorsal approach in either 1% lidocaine and epinephrine infiltration, with/without intravenous sedation or general anaesthesia. The approach was carried between the extensor carpi radialis brevis and longus (ECRB/L). The deep fascia of the ECRB was released, with particular attention to complete release in patients with concomitant lateral epicondylitis. The PIN was decompressed at the level of the arcade of Frohse with release of the proximal 1.5–2 cm of the supinator edge. In cases of concomitant lacertus syndrome, lacertus release followed a technique previously described [15], with a 1.5–2 cm transverse skin incision in the ulnovolar aspect of the proximal forearm.

Return of power was evaluated in all patients either intraoperatively or before discharge from the clinic.

### Outcome measures

Outcome measures were collected preoperatively and via post atsix months postoperatively by an investigator outside the surgical team. A second letter was sent nine months postoperatively if no answer was retrieved after the first. The primary outcomes were the pre- and postoperative quick-DASH with work sub-scores. The DASH questionnaire is a validated tool for standard assessment of the impact on the function of various musculoskeletal diseases and injuries in the upper extremity. Scores range from 0 to 100, with higher ranking indicating worse symptoms [16]. Secondary outcomes included the postoperative VAS for pain, numbness, and subjective satisfaction with the surgery (VAS 0–10) [12].

### Statistical analyses

Statistical analysis was performed using Statistical Package for the Social Sciences for Windows (SPSS version 31.0, Chicago, Illinois, USA). Results were presented as means, median and interquartile ranges. Categorical variables were presented as numbers and percentages. After assessing data distribution, normally distributed continuous variables were compared using Student’s t-test, whereas non-normally distributed continuous variables were analyzed with the Mann–Whitney U test. A p-value of < 0.05 was considered statistically significant.

## Results

### Demographics

A total of 157 arms were operated on 143 patients. Of the whole cohort, 48 were women (33.6%). The average age at the time of surgery was 45.9 years (median 47, range 22 to 67). The dominant arm was operated on in 72.6% of patients while fourteen patients had bilateral surgery. High intensity arm use during work or leisure activities was reported in 69.2% of the cohort, of which 54 were manual labourers and 31 were professional/high-level recreational athletes (21.6%) (Table 1).

**Table 1:** Baseline demographic data of the patient cohort

	Patients *n* = 143	Arms operated *n* = 157
**Age** Mean (Range)	45.9 (22 to 67)	
**Sex** Men Women	95 (66.4%) 48 (33.6%)	
**Side**		
Dominant		114 (72.6%)
Non-dominant		29 (18.5%)
Bilateral		14 (8.9%)
**High-intensity arm use (work/leisure)** Yes No	99 (69.2%) 44 (30.8%)	
**Manual labourers** Yes No	54 (37.8%) 89 (62.2%)	
**Athletes** Yes No	31 (21.6%) 112 (78.4%)	

**Fig. 1:**
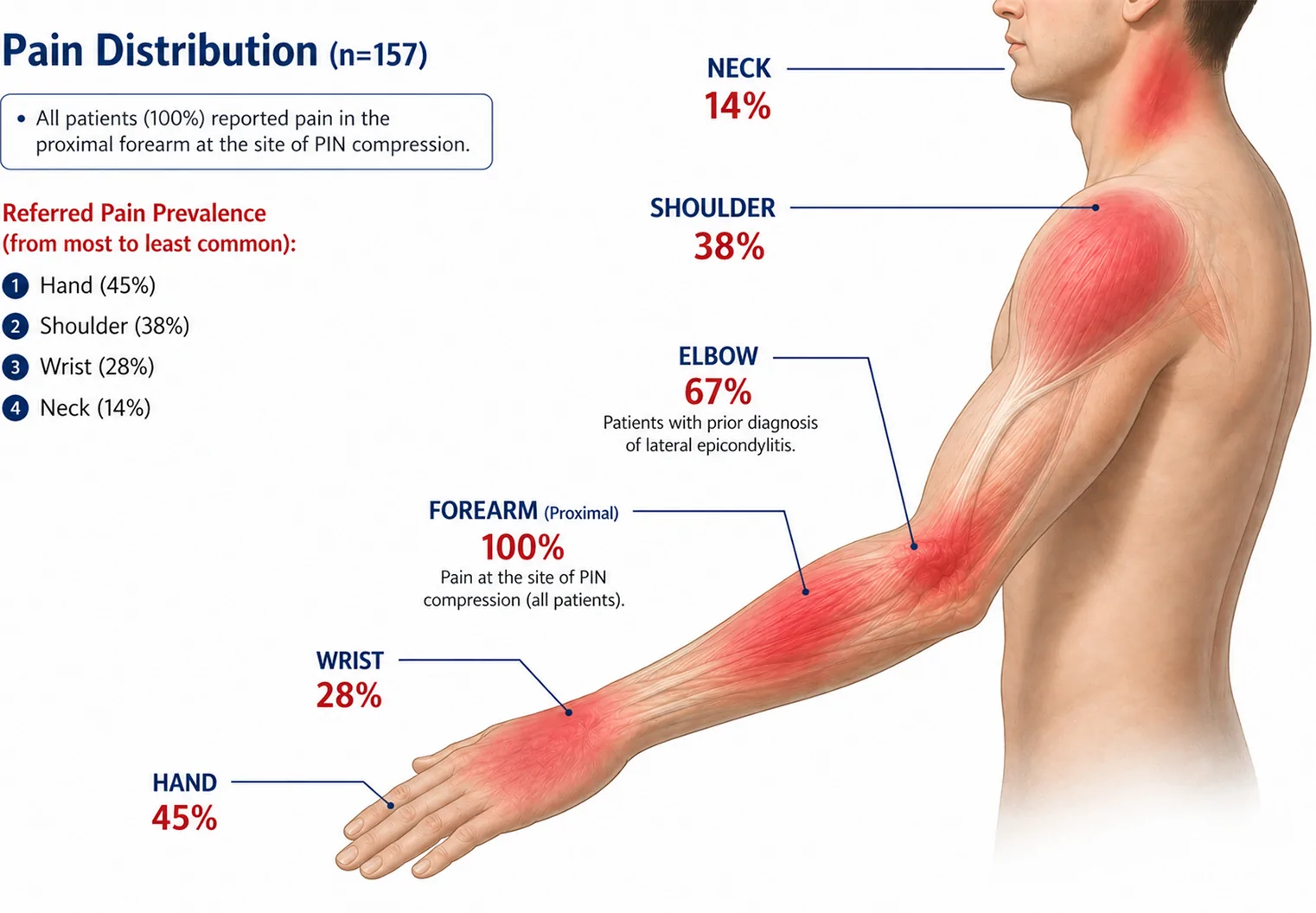
Pain distribution in the cohort of 157 operated arms. All patients reported pain in the dorsal proximal forearm. All patients with a prior diagnosis of lateral epicondylitis (67%) reported lateral elbow pain. Other referred pain sites were, in order of prevalence: Hand (45%); Shoulder (38%); Wrist (28%); and Neck (14%)

All patients complained of pain in the proximal forearm, at the area of the PIN compression. All patients with prior diagnosis of lateral epicondylitis (66.9%) reported pain in the elbow. Referred pain was additionally noted in order from most to least common: hand (45%), shoulder (38%), wrist (28%), neck (14%) (Fig. 1).

One-hundred and five of the treated arms (66.9%) had been previously diagnosed as a “tennis elbow” or lateral epicondylitis. Out of these, 102 had received prior conservative management for lateral epicondylitis with failed treatment effect. The average duration from diagnosis of lateral epicondylitis to surgery was 25 months (median 14, range 2 to 150). Eleven patients had been diagnosed with medial epicondylitis, eight with exertional compartment syndrome while only 14 patients had received no prior diagnosis. The radial tunnel (PIN at the arcade of Frohse) was released in all patients. In 33 arms (21%), the PIN release was done as a solitary procedure. In 114 arms (72.6%) a concomitant lacertus release (proximal median nerve at the lacertus fibrosus) was performed. Four patients had concomitant carpal tunnel release and 6 patients concomitant cubital tunnel release (Table 2).

**Table 2:** Previous diagnoses and surgical procedures of the study cohort

	Arms *n* = 157
**Previous diagnoses**	
No prior diagnosis	14 (8.9%)
Tennis elbow/Lateral epicondylitis	105 (66.9%)
Medial epicondylitis	11 (7.0%)
Exertional compartment syndrome	8 (5.1%)
Elbow trauma	10 (6.4%)
Osteoarthritis	3 (1.9%)
Cervical spine surgery	6 (3.8%)
**Surgical procedures**	
PIN release alone	33 (21.0%)
PIN release & lacertus release	114 (72.6%)
PIN release & carpal tunnel release	4 (2.5%)
PIN release & cubital tunnel release	6 (3.8%)

**Fig. 2:**
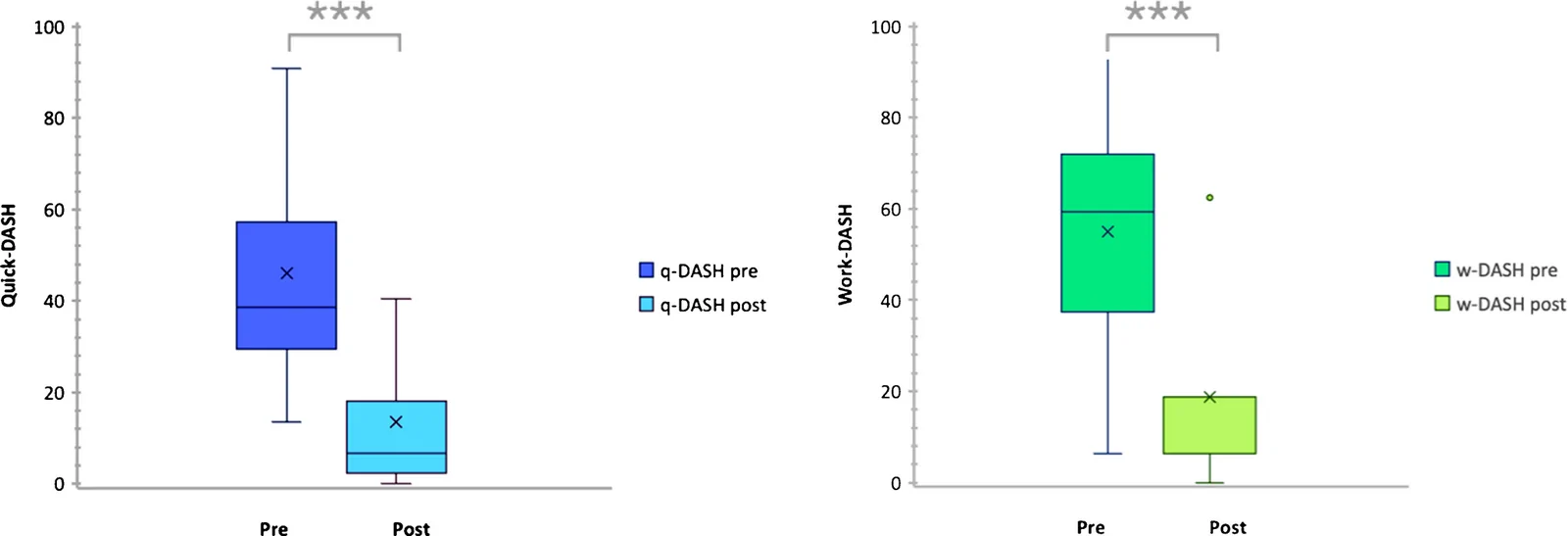
Box-and-whisker plots showing the results of preoperative and postoperative quick-DASH and work-DASH scores. The boxes show the median and interquartile range, and the whiskers show the 25th and 75.^th^ percentiles. (x) marks the average, and (o) indicates outliers outside the whiskers. *** = statistically significant changes in pre- and postoperative scores *(p* < 0.001)

Preoperative quick-DASH with work sub-scores were retrieved from 102 patients operated on between 2012–2019. The PROMs collected from Jan 2020-June 2021 were unfortunately lost as part of computer malware and unavailable for analysis. The 102 collected preoperative scores represented 81% of the treated arms during 2012–2019 (*n* = 126). Of the 102 collected preop PROMs, 66 patients (64.7%) completed the postoperative follow-up questionnaires at a minimum of six months postop. The average preoperative quick DASH was 43.4 (range 13.6 to 90.9) and average work DASH 56.2 (range 6.25 to 100). The average postoperative quick DASH was 11.0 (range 0.0 to 40.5), showing a statistically significant reduction (*p* < 0.001). Similarly, the work DASH sub-scores were also significantly reduced (*p* < 0.001) to 11.2 (range 0.0 to 62.5) (Fig. 2).

**Fig. 3:**
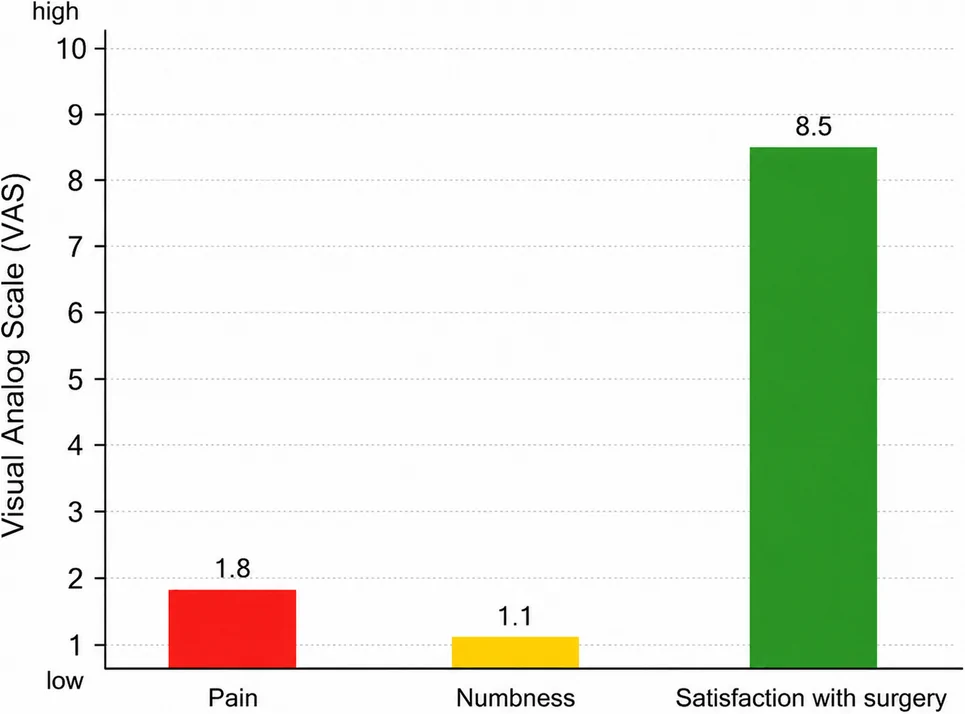
Postoperative Visual Analogue Scale (VAS) scores of Pain, Numbness and Satisfaction with surgical outcome, at minimum 6-month follow-up. Likert Scale 0–10. Score zero represents no pain, no numbness or complete dissatisfaction with surgery. Score 10 represents severe pain, severe numbness or complete satisfaction with surgery

The mean postoperative VAS scores were 1.8 for pain, 1.1 for numbness, and 8.5 for satisfaction with the surgical outcome. Eighty-three percent of patients reported good/excellent satisfaction with the surgical outcomes (Fig. 3).

## Discussion

The most important finding of this study is that patients presenting with chronic lateral elbow and proximal forearm pain in the setting of clinically diagnosed PIN compression frequently demonstrated a broader pattern of upper-extremity dysfunction extending beyond an isolated radial tunnel syndrome. All patients reported pain localized to the proximal forearm at the site of PIN compression, while two-thirds had previously been diagnosed and unsuccessfully treated for lateral epicondylitis. Referred symptoms involving the hand, shoulder, wrist, and neck were common. Based on these observations, we propose the Neurogenic Elbow Disharmony Loop (NEDL) as a hypothesis-generating model in which repetitive stress, multi-site nerve compression, neurogenic weakness, and compensatory biomechanics may interact to perpetuate refractory elbow and proximal forearm pain. Remarkably, the neurogenic basis for this presentation was first proposed more than 140 years ago [17, 18]. The frequent coexistence of lacertus syndrome, the broad distribution of referred symptoms, and the substantial functional improvement observed following surgical decompression suggest that refractory elbow pain may often reflect a more complex neurogenic–biomechanical disorder than traditionally appreciated.

Chronic lateral elbow and proximal forearm pain are frequently attributed to lateral epicondylitis, yet the debate over whether lateral elbow pain arises from tendon, nerve, or both has been ongoing since the condition was first described. In 1882, Morris coined the term “Lawn Tennis Arm”, recognizing both tendinous and neural contributions to the syndrome [18, 19]. The neurogenic argument was advanced the very following year by Winkworth [17], attributing lateral elbow pain specifically to compression of “the posterior interosseous nerve as it passes through the substance of the supinator brevis muscle and the median nerve as it passes between the two heads of the pronator radii teres muscle”—in essence, anticipating the anatomical basis of the NEDL by more than a century. Despite this prescient observation, the neurogenic hypothesis was largely eclipsed by the tendinopathy model that dominated throughout the twentieth century. Today, RTS and PIN compression remain a primary differential that is frequently overlooked [2, 20]. Cutts et al. [20] estimated that approximately 30% of cases diagnosed as tennis elbow may involve PIN entrapment. While standard management for lateral epicondylitis focuses on the common extensor tendon through conservative measures or localised debridement, these interventions often fail to provide lasting relief if the underlying aetiology is neurogenic [2, 21]. This is reflected in the current cohort, where 66.9% of patients had a prior diagnosis of lateral epicondylitis and 102 had failed extensive conservative treatment, highlighting the diagnostic confusion that persists when nerve entrapment at the arcade of Frohse presents with overlapping symptomatic patterns.

It is striking that Winkworth’s 1883 formulation described compression of both the posterior interosseous nerve and proximal median nerve [17], precisely the two entrapment patterns that form the basis of the NEDL and accounted for the majority of procedures in the present series. The intervening 140 years have brought substantial advances in nerve physiology, imaging, and outcome measurement; however, the fundamental anatomical observation that lateral elbow pain may reflect dual-site neurogenic dysfunction remains highly relevant. That the present study, following 157 patients after PIN decompression, arrives at a similar conclusion underscores the continued importance of careful clinical examination, particularly in conditions where electrodiagnostic and imaging studies may be inconclusive.

RTS remains a controversial diagnosis, with a reported incidence of only 0.003–0.03% [2, 22]. A key reason for its frequent under-recognition is that conventional electrodiagnostic studies and imaging are typically unremarkable, as low-grade or dynamic PIN compression presents as a Sunderland 0 ischaemic conduction block that does not produce axonal degeneration or demyelination detectable by standard nerve conduction studies [6, 7, 22]. In the present cohort, electrodiagnostic studies were rarely performed and invariably negative or inconclusive, underscoring that RTS is fundamentally a clinical diagnosis. The diagnostic framework applied in this study was the clinical triad for nerve compressions [4]: (1) weakness on manual muscle testing of the ECU, reflecting PIN compression at the arcade of Frohse; (2) a positive SCT at the level of the radial tunnel; and (3) focal pain on compression at the site of entrapment. The importance of the motor examination is strongly supported by the present findings: return of ECU power on clinical examination was observed in all 157 operated arms either intraoperatively or before discharge, consistent with the rapid resolution of ischaemic conduction block expected following surgical decompression [7, 8].

The complexity of forearm pain is compounded by concomitant entrapments such as lacertus syndrome, carpal tunnel syndrome, and cubital tunnel syndrome [3, 9, 12]. Lacertus syndrome is a dynamic compression of the median nerve at the level of the lacertus fibrosus that is frequently misdiagnosed or overshadowed by distal entrapments such as carpal tunnel syndrome [23, 24]. In the current study, 72.6% of patients required concomitant lacertus release alongside PIN decompression, suggesting that isolated single-site nerve compression is the exception rather than the rule. Although this proportion may appear high, it likely reflects both the referral nature of the practice and the systematic use of a clinical examination protocol specifically designed to identify proximal median nerve compression [25]. These findings support the integration of seemingly distinct pathologies into the NEDL: rather than viewing lateral elbow pain in isolation, the model accounts for the interdependent nature of neurogenic weakness, altered biomechanics, and multi-site compression across the upper limb.

The NEDL proposes that repetitive stress from high-use activities initiates a self-perpetuating cycle of neurogenic and biomechanical dysfunction. One proposed entry point into this cycle is dynamic median nerve compression at the level of the lacertus fibrosus, which may present as a Sunderland 0 ischaemic conduction block [7]. This results in subtle but functionally significant weakness in the flexor group, specifically the FCR, FPL, and FDP II, impairing the scaling and timing of precision grip force [9, 24, 26]. To compensate for reduced grip stability and flexor power, the patient may adopt a wrist-extension strategy that stabilizes the hand during manual tasks and promotes a tenodesis effect facilitating pinch grip [26, 27]. Chronic overload of the lateral elbow may subsequently contribute to ECRB tendinopathy or fascial shortening, as originally described by Roles and Maudsley in their “middle finger extension test” [28], which in turn increases mechanical pressure on the underlying radial tunnel and predisposes the PIN to compression at the arcade of Frohse [1, 20, 29].

**Fig. 4:**
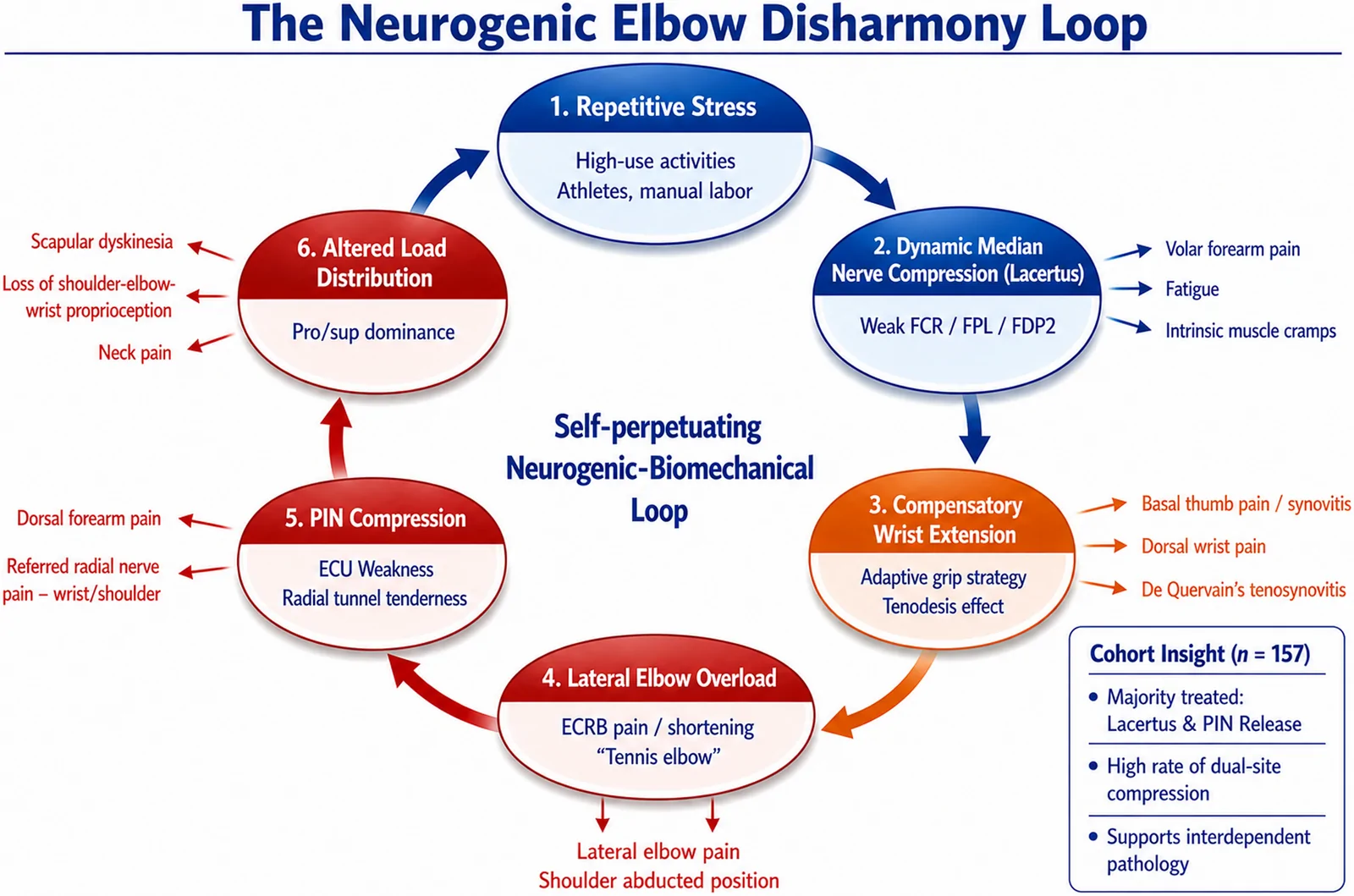
The Neurogenic Elbow Disharmony Loop (NEDL): a self-perpetuating neurogenic–biomechanical model of chronic elbow pain, supported by a surgical cohort of 157 arms. The NEDL proposes six interdependent stages. Repetitive stress from high-use activities initiates dynamic median nerve compression at the lacertus fibrosus (1), producing FCR, FPL, and FDP2 weakness with volar forearm pain and fatigue (2). Loss of flexor grip stability drives compensatory wrist extension and tenodesis-based grip, overloading the lateral elbow (3), and contributing to ECRB tendinopathy and the clinical picture of tennis elbow (4). Increased radial tunnel pressure leads to PIN compression at the arcade of Frohse, with ECU weakness and referred pain to the wrist and shoulder (5). The resulting altered load distribution propagates proximally — generating scapular dyskinesia, proprioceptive loss, and neck pain — closing the loop (6). In the present cohort, 72.6% of patients required combined lacertus and PIN release, consistent with the high rate of dual-site compression predicted by the model. FCR, flexor carpi radialis; FPL, flexor pollicis longus; FDP2, flexor digitorum profundus to the index finger; ECU, extensor carpi ulnaris; ECRB, extensor carpi radialis brevis; PIN, posterior interosseous nerve

The resulting PIN entrapment may lead to ECU weakness and persistent dorsal proximal forearm pain, completing a state of “disharmony” characterised by pronation-supination dominance [30]. This altered load sharing eventually propagates proximally up the kinetic chain, as seen in the 38% of patients reporting shoulder pain and 14% reporting neck stiffness, a pattern that mirrors the multi-site symptom clusters described in broader human disharmony models [13, 31] (Fig. 4). The significant functional improvement following comprehensive multi-site decompression, and the high satisfaction rates observed in this cohort, indicate that breaking the loop at multiple points of entrapment is essential for resolving refractory chronic elbow pain and restoring functional endurance.

Surgical decompression for RTS has historically been regarded as yielding inconsistent outcomes, and the results in the literature have indeed been variable. In the most comprehensive systematic review to date, Raymond et al. [2] reported that postoperative satisfaction across published RTS surgical cohorts ranged from as low as 39% to over 80%, with the highest satisfaction rates consistently observed in studies employing a dorsal surgical approach. Volar approaches, by contrast, were associated with lower satisfaction, higher complication rates, and poorer functional scores. To our knowledge, the present cohort represents one of the largest series of PIN decompressions reported in the literature, and all cases were performed through a dorsal approach with release at the arcade of Frohse. Our outcomes align with the upper range reported by Raymond et al. [2]: QuickDASH improved by more than 30 points (43.4 to 11.0, *p* < 0.001), and 83% of patients rated their outcome as good or excellent, comparable to the best results achieved in dorsal-approach series. These findings are consistent with previous literature reporting favorable outcomes following a dorsal approach to PIN decompression and suggest that surgical outcomes may be improved when the diagnosis is established through a structured clinical assessment and all clinically relevant sites of nerve entrapment are addressed.

Several limitations of this study deserve acknowledgement. There was no control group, which limits comparisons with continued conservative treatment, isolated lateral epicondylopathy management, or single-site decompression. Most patients underwent combined procedures, making it difficult to isolate the independent contribution of PIN decompression. The diagnosis of RTS and associated entrapments was primarily clinical, based on manual muscle testing, focal tenderness, and SCT. Whilst this reflects contemporary peripheral nerve practice, these tests remain examiner-dependent. Preoperative and postoperative PROMs were not available for all patients, partly because of loss of data related to computer malware. Finally, the NEDL should be interpreted as a hypothesis-generating pathophysiologic model rather than a proven causal mechanism. Despite these limitations, this study provides clinical support for a broader neurogenic–biomechanical interpretation of refractory lateral elbow and proximal forearm pain and introduces the NEDL as a hypothesis-generating framework that may guide future prospective investigation.

## Conclusion

Surgical decompression of posterior interosseous nerve (PIN) compression at the Arcade of Frohse, diagnosed by the clinical triad of ECU weakness, a positive sensory collapse test, and focal tenderness at the entrapment site, resulted in significant functional improvement and high patient satisfaction. The high prevalence of concomitant lacertus syndrome, together with the characteristic bidirectional pattern of referred symptoms extending both distally and proximally, supports the Neurogenic Elbow Disharmony Loop (NEDL) as a hypothesis-generating model linking dynamic median and radial nerve compression to chronic lateral elbow pain. These findings highlight the importance of evaluating patients with refractory elbow pain for multiple sites of nerve entrapment and considering a comprehensive treatment strategy that addresses the entire neuro-musculoskeletal kinetic chain rather than a single anatomical pain generator.

## Data Availability

The data underlying this article are available in the article.

## Abbreviations

*DASH*: Disability of the Arm, Shoulder and Hand Questionnaire
*ECU*: Extensor carpi ulnaris
*ECRB*: Extensor carpi radialis brevis
*FCR*: Flexor carpi radialis
*FDP II*: Flexor digitorum profundus to the index finger
*FPL*: Flexor pollicis longus
*NEDL*: Neurogenic Elbow Disharmony Loop
*PIN*: Posterior interosseous nerve
*RTS*: Radial tunnel syndrome
*SCT*: Scratch collapse test/Sensory collapse test
*VAS*: Visual analogue scale

## References

[1] Speers CJ, Bhogal GS, Collins R (2018) Lateral elbow tendinosis: a review of diagnosis and management in general practice. Br J Gen Pract 68(676):548–549 30361321 10.3399/bjgp18X699725 PMC6193783

[2] Raymond B, Cueto RJ, Mazudie Ndjonko LC, Hao KA, Pfaehler CD, Buchanan TR et al (2026) Clinical outcomes of operative management for radial tunnel syndrome according to surgical approach: a systematic review. HAND 21(2):176–185 39921571 10.1177/15589447251315761 PMC11807271

[3] Hagert E, Lalonde D (2017) Nerve entrapment syndromes. In: Chang J, Neligan PC (eds) Neligan’s plastic surgery: hand and upper limb, vol vol 6. Elsevier Health Sciences, pp 525–548

[4] Hagert E, Hagert CG (2014) Upper extremity nerve entrapments. Plast Reconstr Surg 134(1):71–80 24622568 10.1097/PRS.0000000000000259

[5] Lawand JJ, Saab D, Luan A, Curtin C, Hagert E (2025) Return to play and outcomes of surgically treated upper limb nerve entrapment in athletes: a systematic review. Int Orthop 49(4):871–880 40021549 10.1007/s00264-025-06473-9

[6] Pan D, Patel AU, Patterson JM, Johnson AR, Gordon T, Mackinnon SE (2025) Surgeons, physiatrists and neurologists speaking “EDX and nerve surgery”: a paradigm shift for nerve injured patients. JPRAS Open 47:3–20 41497893 10.1016/j.jpra.2025.11.004 PMC12766253

[7] Pripotnev S, Llaneras NS, Teixeira B, Lee E, Patterson M, Seu M et al (2025) The classification of nerve injury revisited: Sunderland 0-VI. Plast Surg. 10.1177/22925503251375862 PMC12550730 41141462

[8] Peters BR, Pripotnev S, Chi D, Mackinnon SE (2022) Complete foot drop with normal electrodiagnostic studies: sunderland “Zero” ischemic conduction block of the common peroneal nerve. Ann Plast Surg 88(4):425–428 34864748 10.1097/SAP.0000000000003053

[9] Hagert E, Curtin C (2023) Median and ulnar nerve compressions: simplifying diagnostics and surgery at the elbow and hand. Plast Reconstr Surg 152(1):155e-e165 37382919 10.1097/PRS.0000000000010268

[10] Patel AU, Biaggi-Ondina A, Mackinnon SE (2026) Commentary: quantitative scratch collapse test methodology with handheld dynamometer: normative upper-extremity force data and reliability analysis. J Hand Surg Glob Online 8(3):100995 42130909 10.1016/j.jhsg.2026.100995 PMC13161687

[11] McCarthy JE, Attaluri P, Nicksic P (2024) On the physiology of the sensory-collapse test. J Hand Surg Am 49(6):603–606 38456864 10.1016/j.jhsa.2024.01.017

[12] Hagert E, Jedeskog U, Hagert CG, Marín FT (2023) Lacertus syndrome: a ten year analysis of two hundred and seventy five minimally invasive surgical decompressions of median nerve entrapment at the elbow. Int Orthop 47(4):1005–1011 36757413 10.1007/s00264-023-05709-w PMC10014674

[13] Sharma K, Friedman JM (2025) The human disharmony loop: a case series proposing the unique role of the pectoralis minor in a unifying syndrome of chronic pain, neuropathy, and weakness. J Clin Med. 10.3390/jcm14051769 40095905 PMC11901267

[14] Friedman JM, Iyengar J, Sharma K (2025) Validation of the human disharmony loop: pectoralis minor tenotomy significantly reduces pain and improves function in historically challenging patients who meet reproducible and explicit diagnostic criteria. PLoS ONE 20(10):e0326815 41160656 10.1371/journal.pone.0326815 PMC12571246

[15] Hagert E (2013) Clinical diagnosis and wide-awake surgical treatment of proximal median nerve entrapment at the elbow: a prospective study. Hand (N Y) 8(1):41–46 24426891 10.1007/s11552-012-9483-4 PMC3574476

[16] Gummesson C, Ward MM, Atroshi I (2006) The shortened disabilities of the arm, shoulder and hand questionnaire (QuickDASH): validity and reliability based on responses within the full-length DASH. BMC Musculoskelet Disord 7:44 16709254 10.1186/1471-2474-7-44 PMC1513569

[17] Winckworth CE (1883) Lawn-tennis elbow. BMJ 2:708

[18] Morris H (1882) The rider’s sprain. Lancet 120(3074):133–134

[19] Thurston AJ (1998) The early history of tennis elbow: 1873 to the 1950s. Aust N Z J Surg 68(3):219–224 9563455 10.1111/j.1445-2197.1998.tb04751.x

[20] Cutts S, Gangoo S, Modi N, Pasapula C (2020) Tennis elbow: a clinical review article. J Orthop 17:203–207 31889742 10.1016/j.jor.2019.08.005 PMC6926298

[21] Keles A, Palamar D, Gunduz A, Akarirmak U (2026) Ultrasonographic and electrophysiologic assessment of radial tunnel syndrome in patients with treatment-resistant lateral epicondylitis: insights into diagnostic utility and clinical implications. Am J Phys Med Rehabil 105(4):330–337 41082715 10.1097/PHM.0000000000002877

[22] Patterson JMM, Medina MA, Yang A, Mackinnon SE (2024) Posterior interosseous nerve compression in the forearm, AKA radial tunnel syndrome: a clinical diagnosis. Hand (N Y) 19(2):228–235 36082441 10.1177/15589447221122822 PMC10953526

[23] Al-Hashimi Y, Ferembach B, Martinel V, Hagert E (2024) Painful nerve compression beyond the carpal tunnel: recognizing the lacertus syndrome. Plast Aesthet Res 11(9):1–10

[24] Lalonde D (2015) Lacertus syndrome: a commonly missed and misdiagnosed median nerve entrapment syndrome. BMC Proc 9(S3):A74

[25] Hagert E (2025) The clinical triad: a structured approach to diagnosing peripheral nerve compressions. Int Orthop 49(4):899–909 40053068 10.1007/s00264-025-06452-0 PMC11971183

[26] Dierick F, Brismée JM, White O, Bouché AF, Périchon C, Filoni N et al (2021) Fine adaptive precision grip control without maximum pinch strength changes after upper limb neurodynamic mobilization. Sci Rep 11(1):14009 34234161 10.1038/s41598-021-93036-8 PMC8263565

[27] Cowley JC, Leonardis J, Lipps DB, Gates DH (2017) The influence of wrist posture, grip type, and grip force on median nerve shape and cross-sectional area. Clin Anat 30(4):470–478 28281294 10.1002/ca.22871

[28] Roles NC, Maudsley RH (1972) Radial tunnel syndrome: resistant tennis elbow as a nerve entrapment. J Bone Joint Surg Br 54(3):499–508 4340924

[29] Bonczar M, Ostrowski P, Dziedzic M, Kasprzyk M, Obuchowicz R, Zacharias T et al (2023) Evaluation of lateral epicondylopathy, posterior interosseous nerve compression, and plica syndrome as co-existing causes of chronic tennis elbow. Int Orthop 47(7):1787–1795 37071147 10.1007/s00264-023-05805-x PMC10267267

[30] Ceri T, Podda A, Behr J, Brumpt E, Alilet M, Aubry S (2019) Posterior interosseous nerve of the elbow at the arcade of Frohse: ultrasound appearance in asymptomatic subjects. Diagn Interv Imaging 100(9):521–525 30935861 10.1016/j.diii.2019.03.007

[31] Sharma K, Iyengar J, Friedman J (2025) The human disharmony loop: the anatomic source behind subacromial impingement and pain. J Clin Med 14(16) 10.3390/jcm14165650 PMC12387092 40869475

